# Untargeted fecal metabolome analysis in obese dogs after weight loss achieved by feeding a high-fiber-high-protein diet

**DOI:** 10.1007/s11306-021-01815-1

**Published:** 2021-07-06

**Authors:** Sandra Bermudez Sanchez, Rachel Pilla, Benjamin Sarawichitr, Alessandro Gramenzi, Fulvio Marsilio, Joerg M. Steiner, Jonathan A. Lidbury, Georgiana R. T. Woods, Jan S. Suchodolski, Alexander J. German

**Affiliations:** 1 Gastrointestinal Laboratory, College of Veterinary Medicine and Biomedical Sciences, Texas A&M University, College Station, TX USA; 2 Veterinary Medicine Sciences, University of Teramo, Teramo, Italy; 3 Institute of Life Course and Medical Sciences, University of Liverpool, Leahurst, Neston, UK

**Keywords:** Canine obesity, Fecal microbiota, Fecal metabolome, Weight loss, Untargeted metabolome analysis

## Abstract

**Introduction:**

In humans and companion animals, obesity is accompanied by metabolic derangements. Studies have revealed differences in the composition of the fecal microbiome between obese dogs and those with an ideal body weight.

**Objectives:**

We have previously reported that the fecal microbiome in obese dogs changes after controlled weight reduction, induced by feeding a diet high in fiber and protein. Despite these findings, it is unclear if taxonomic differences infer differences at the functional level between obese dogs and those with an ideal body weight.

**Methodology:**

Untargeted fecal metabolome analysis was performed on dogs with obesity before and after weight loss achieved by feeding a high-fiber-high-protein diet.

**Results:**

Fecal metabolome analysis revealed a total of 13 compounds that changed in concentration in obese dogs after weight loss. Of these compounds, metabolites associated with bacterial metabolism decreased after weight loss including purine, L-(-)-methionine, coumestrol, and the alkaloids 1-methylxanthine and trigonelline. Conversely, the polyphenols (-)-epicatechin and matairesinol and the quinoline derivatives 1,5-isoquinolinediol and 2-hydroxiquinoline increased after weight loss.

**Conclusion:**

These results suggest differences in intestinal microbiome at the functional level after weight loss, but further studies are needed to determine the role of these compounds in the etiology of obesity and weight loss.

**Supplementary Information:**

The online version contains supplementary material available at 10.1007/s11306-021-01815-1.

## Introduction

Canine obesity has recently been characterized as a disease by many international veterinary organizations (Ward et al., 2019), on account of its significant impact on health and wellbeing (German, 2006; German et al., 2010). Recent studies have identified metabolic changes associated with canine obesity, including insulin resistance, decreased adiponectin concentrations and markers of renal function or disease (German et al., 2009; Tvarijonaviciute et al., 2012, 2013). Other obesity-related metabolic changes have been identified using proteomic analysis, including altered expression of proteins involved in lipid metabolism, immune response, and antioxidant status (Tvarijonaviciute et al., 2016).

Obesity is associated with alterations of the gut microbiota (Ridaura et al., 2013; Turnbaugh et al., 2009). The first studies that compared the fecal microbiome of obese dogs and those with an ideal body weight showed only minor differences in their fecal microbiome (Handl et al., 2013; Park et al., 2015). However, subsequent studies evaluating the fecal microbiota of obese dogs before and after weight loss, reported an increase in the bacterial biodiversity and a decrease of the Firmicutes/Bacteroidetes ratio after the dogs completed their weight loss program (Bermudez Sanchez et al., 2020; Salas-Mani et al., 2018).

Gut bacteria are known to have important functions affecting host physiology (Sittipo et al., 2019) and maintaining gut homeostasis, including development and modulation of the immune system, the gut epithelium, bile acid metabolism, xenobiotic degradation, and biosynthesis of vitamins (Jandhyala et al., 2015). In addition, the gut microbiota harbors an expansive number of genes that confer upon bacteria a wide variety of digestive functions, associated with dietary and host metabolites (Kho & Lal, 2018).

Studies using animal models that focused on defining the role of the gut microbiota in the etiology of obesity, point to a direct interaction with the host through bacterial metabolites (Boulangé et al., 2016). In this respect, the gut microbiota are thought to modulate host gut microenvironment and host energy homeostasis by metabolizing nutrients from the diet (Arora et al., 2011). Examples include synthesis of short-chain fatty acids (SCFAs) from undigested polysaccharides and bile acid interconversion (Rowland et al., 2018). These microbial processes are thought to contribute to the etiology of obesity by modulation of energy generation, storage, and expenditure, in addition to intestinal inflammation mediated by LPS (Harakeh et al., 2016).

The analysis of the fecal metabolome is a non-invasive method of characterizing functional differences of the gut microbiome between individuals, with results depending upon the composition of the overall gut microbiota (Zierer et al., 2018). However, the vast majority of metabolites remain unknown with undefined functions. Therefore, to better understand associations between the gut microbiota and obesity in dogs, we evaluated the composition of the fecal metabolome in 20 client-owned dogs with obesity, that had recently participated in a study examining the composition of the fecal microbiota (Bermudez Sanchez et al., 2020). Untargeted fecal metabolome analysis was performed on fecal samples of obese dogs before and after controlled weight reduction by feeding them a diet rich in protein and fiber.

## Material and methods

### Study animals, eligibility criteria, and ethical considerations

Dogs with obesity enrolled into this study had been referred to the Royal Canin Weight Management Clinic at the University of Liverpool, UK, between June 2009 and August 2017. All dogs had completed their weight loss program between November 2009 and August 2018, and eligibility criteria were the same as those described previously (Bermudez Sanchez et al., 2020), including having a body condition score (BCS) > 6 on a 9-point scale, no antimicrobial usage in the past month (Igarashi et al., 2014), and no signs of gastrointestinal disease. The studied group comprised 25 dogs with obesity. Five dogs stopped the study prematurely and were excluded from the analysis; the remaining 20 dogs completed the weight loss programme, and fecal samples were available before dietary change (T0) and after a period of controlled weight reduction with a therapeutic diet (see below) to reach a pre-defined target weight (T1).

The study protocol was reviewed and approved by the University of Liverpool Veterinary Research Ethics Committee (Approval reference: RETH000353 and VREC793), the Royal Canin ethical review committee, and the WALTHAM ethical review committee. Owners of dogs with obesity gave informed consent in writing.

### Weight loss regimen

Full details of the weight loss regimen have been previously described (German et al., 2015). Briefly, dogs were first determined to be systemically well (apart from their obesity), based on physical examination findings, complete blood count, serum biochemical analysis, serum free thyroxine concentration (by equilibrium dialysis), and urinalysis. Body condition score (BCS) was estimated using a 9-integer scale (Laflamme, 1997) by the attending clinician (AJG). Body composition was measured by dual-energy x-ray absorptiometry (DEXA), as previously described (Raffan et al., 2006), the results of which were used to determine the ideal body weight of each dog (German et al., 2015). Body composition measurements were available for 19 of the 20 dogs, from both before and after weight reduction.

All dogs were fed either a dry or wet high-protein high-fiber therapeutic weight reduction diet (Canine Satiety® diet, Royal Canin), with an increased protein and fiber content as previously described (German et al., 2015). Advice was also given to owners about lifestyle and activity alterations that could help their dog lose weight. For each dog, a target weight was also set, which was individually tailored for each dog, based upon expected health benefits, once again, as described (German, 2016). The weight reduction process was monitored through regular weight checks using calibrated electronic scales.

### Fecal collection

As previously described (Bermudez Sanchez et al., 2020), fecal samples were collected after spontaneous defecation and stored at − 20 °C, before being shipped to the Gastrointestinal Laboratory at Texas A&M University in February 2019.

### Sample preparation for metabolome analysis

Samples were lyophilized, weighed, and extracted with a methanol:chloroform:water based extraction method. Briefly 800 µL ice cold methanol:chloroform (1:1, v:v) was added to each sample in a bead based lysis tube (Bertin, Rockville, MD). Samples were extracted on a Precyllys 24 (Bertin) tissue homogenizer for 30 s at a speed of 6000 rpm. The supernatant was collected and samples were homogenized a second time with 800 µL ice cold methanol:chloroform. Then 600 µL ice cold water was added to the combined extract, vortexed, and centrifuged to separate the phases. The upper aqueous layer was passed through a 0.2 µm nylon filter (Merck Millipore, Burlington, MA). Then 500 µL of the filtered aqueous phase was passed through a 3 kDa cutoff column (Thermo Scientific) and the flow through was collected for analysis.

### Metabolome analysis by HPLC–MS

Untargeted liquid chromatography high resolution accurate mass spectrometry (LC-HRAM) analysis was performed on a Q Exactive Plus orbitrap mass spectrometer (Thermo Scientific, Waltham, MA) coupled to a binary pump HPLC (UltiMate 3000, Thermo Scientific). Full MS spectra were obtained at 70,000 resolution (200 m/z) with a scan range of 50–750 m/z. Full MS followed by ddMS2 scans were obtained at 35,000 resolution (MS1) and 17,500 resolution (MS2) with a 1.5 m/z isolation window and a stepped NCE (20, 40, 60). Samples were maintained at 4 °C before injection. The injection volume was 10 µL. Chromatographic separation was achieved on a Synergi Fusion 4 µm, 150 mm × 2 mm reverse phase column (Phenomenex, Torrance, CA) maintained at 30 °C using a solvent gradient method. Solvent A was water (0.1% formic acid). Solvent B was methanol (0.1% formic acid). The gradient method used was 0–5 min (10% B to 40% B), 5–7 min (40% B to 95% B), 7–9 min (95% B), 9–9.1 min (95% B to 10% B), 9.1–13 min (10% B). The flow rate was 0.4 mL/min. Sample acquisition was performed by the Xcalibur software package (Thermo Scientific). Data analysis was performed with the Compound Discoverer software package 2.1 (Thermo Scientific).

### Data analysis (Compound Discoverer 2.1)

Background was excluded by setting the minimal peak intensity as greater than 169,436.00. As inclusion criteria for the analysis, we applied at least an 80% of match score with the compound of reference provided by the MzCloud library, which considers the mass spectrum of a catalog of the compounds. Unnamed and repeated compounds were excluded. Resulting peaks were manually curated, and the peak intensity for each sample and compound was exported into an excel file (Microsoft Excel; Supplementary data 1). A total of 134 named compounds were identified in the fecal samples of the dogs enrolled.

### Statistical analysis

Normality was tested using Shapiro–Wilk tests for all continuous variables. Results were reported as median (range) when data were non-parametric, and as mean (± SD) when data passed normality testing. Differences in dog characteristics between groups were compared using *t*-tests or Wilcoxon tests for paired analyses and Mann–Whitney tests for unpaired analyses. Metabolomic data were analyzed with the web-based free tool MetaboAnalyst 5.0.; http://www.metaboanalyst.ca) (Chong & Xia, 2020; Xia et al., 2015). Data were normalized by pareto scaling and log transformation. MetaboAnalyst was used to generate Principal Coordinate Analysis (PCA) and Partial Least Squares-Discriminant Analysis (PLS-DA) plots in 2D to display metabolic communities for all groups. Heatmap visualization was performed based on Wilcoxon tests for paired analyses and reorganization of metabolites to show contrast between the groups. Correction for multiple testing was done by calculating the false discovery rate (FDR). Normalized data were used with a statistical software package, GraphPad Prism version 8.0 (La Jolla, California, USA), to generate graphs for individual values of fecal metabolites for each dog.

## Results

### Animal population characteristics and details of the study period

At enrollment, the median age of the dogs was 63 months (range 16 to 135 months). The breeds of the dogs enrolled in the study were: Labrador Retriever (6), Golden Retriever (2), Cavalier King Charles spaniel (2), Border Collie (1), Lhasa Apso (1), American Bulldog (1), Dachshund (1), Rottweiler (1), Newfoundland (1), Bichon Frise (1), and mixed breed (3). Ten dogs were neutered female and 10 were neutered male. Prior to weight reduction, the median BCS was 8 (range 6 to 9). Mean body fat mass before weight loss was 45% (SD 5.2%).

The median time between fecal sampling (T0 to T1) was 271 (range 112 to 768) days during which time dogs had lost a mean of 26% (SD 8.6%) of their starting bodyweight, equating to a rate of 0.7% (SD 0.30%) per week. Body condition score changed significantly (T0 median BCS, 8 [range 6–9]; T1 median BCS, 5 [range 4–7], *P* < 0.001). Body fat mass decreased significantly after weight loss (T0 mean, 45% [SD 5.2%]; T1 mean, 30% [SD 6.7%], *P* < 0.001). The mean daily energy intake during the weight loss period was 60.8 kcal/kg^0.75^ (SD 5.6 kcal/kg^0.75^) calculated based on the ideal body weight (Bermudez Sanchez et al., 2020).

No significant associations between baseline fecal metabolites and sex as well as age were found (Supplementary data 2).

### The fecal metabolome of dogs with obesity changes after a period of controlled weight reduction

**Fig. 1:**
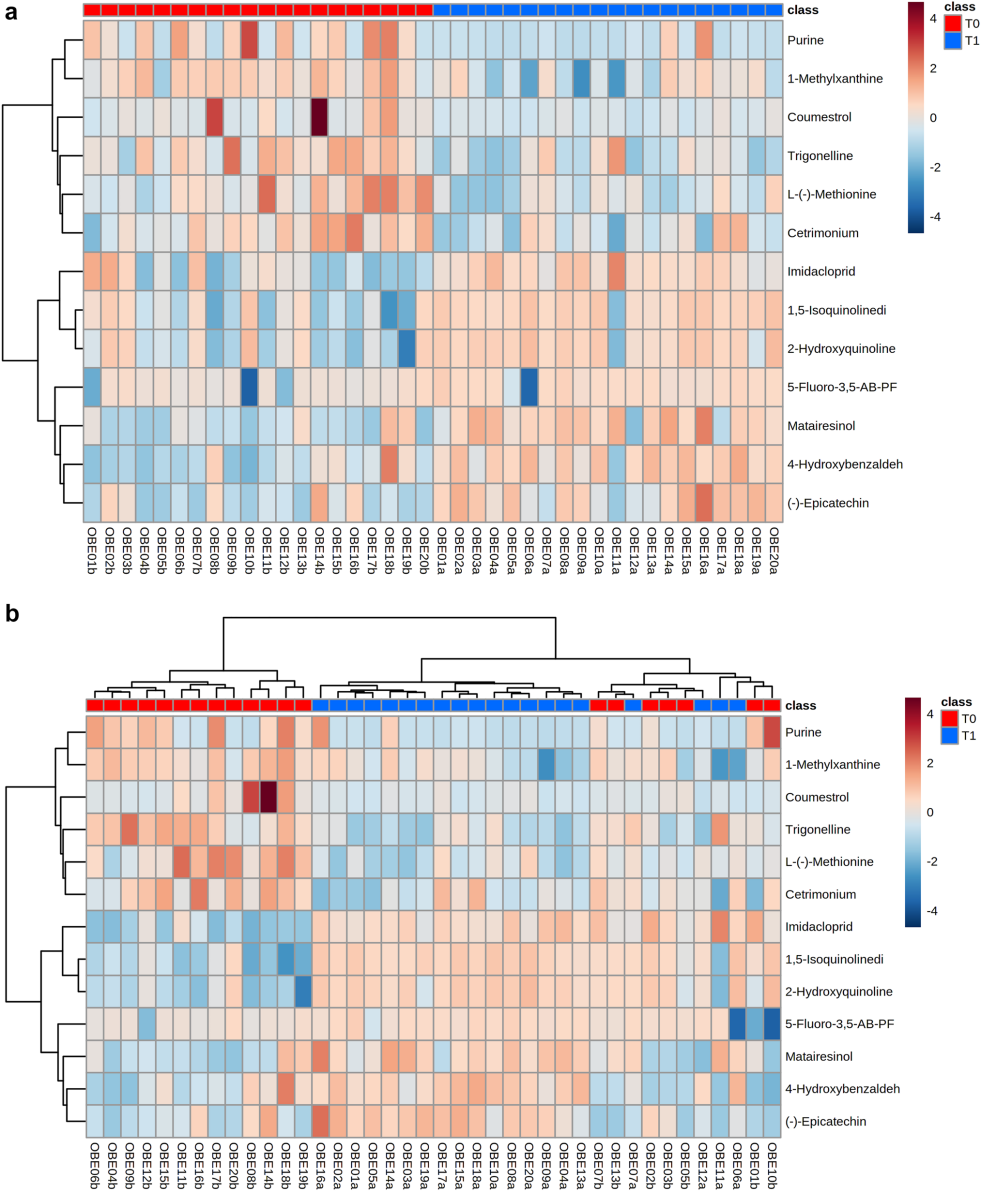
**A** Reorganized and **B** not reorganized hierarchical clustering and heatmaps of the 13 metabolites that were different in their peak intensity before and after weight reduction. Ordered columns represent the fecal metabolite profile for each dog before (T0) and after a period of weight reduction (T1) using a therapeutic diet and sorted by group. Colored bars on the x-axis represent group of dogs (red, obese dogs before weight loss; blue, obese dogs after weight loss). Rows represent the 13 metabolites that differed, highlighting two clusters of metabolites: the first comprised metabolites that decreased after weight reduction (i.e., purine, 1-methylxanthine, coumestrol, trigonelline, L-(-)-methionine, and cetrimonium); the second comprised metabolites that increased after weight reduction (i.e., imidacloprid, 1,5-isoquinolinediol, 2-hydroxiquinoline, 5-fluoro-3,5-AB-PFUPPYCA, matairesinol, 4-hydroxybenzaldehido, and (-)-epicatechin)

A total of 134 compounds were identified in the feces of the dogs, of which, 13 metabolites changed significantly between T0 and T1, based on adjusted *P*-values (Fig. 1).

**Fig. 2:**
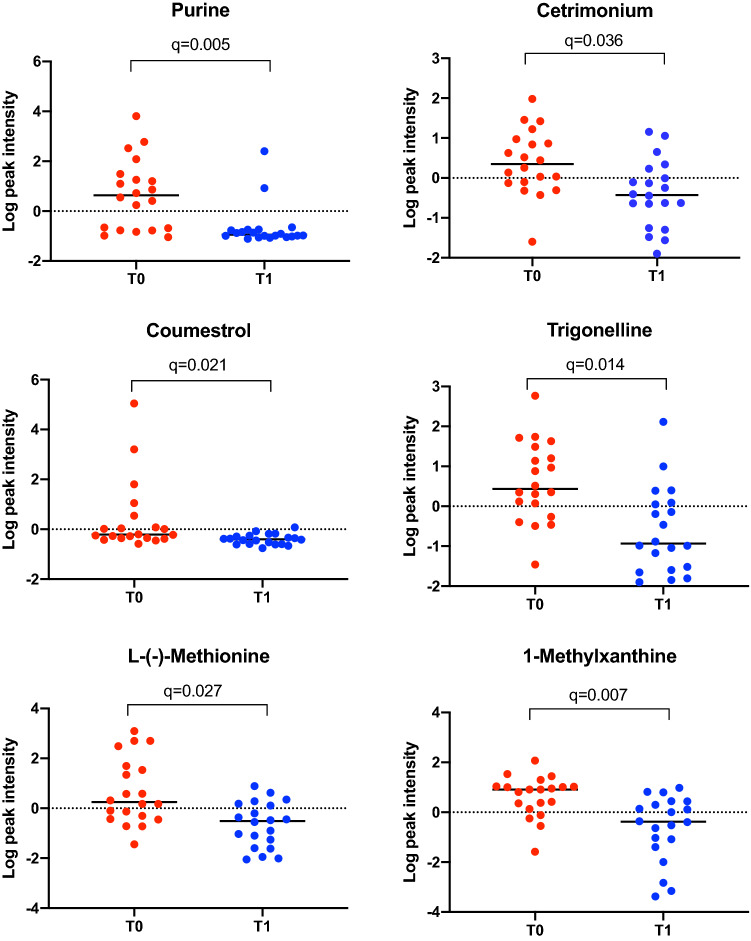
Normalized data were used to create individual value graphs of the fecal metabolites that decreased after weight reduction in dogs. The bar represents the median for each group, (T0) before, (T1) after a period of weight reduction. Wilcoxon tests were used for statistical comparison and significance was based on FDR values

**Fig. 3:**
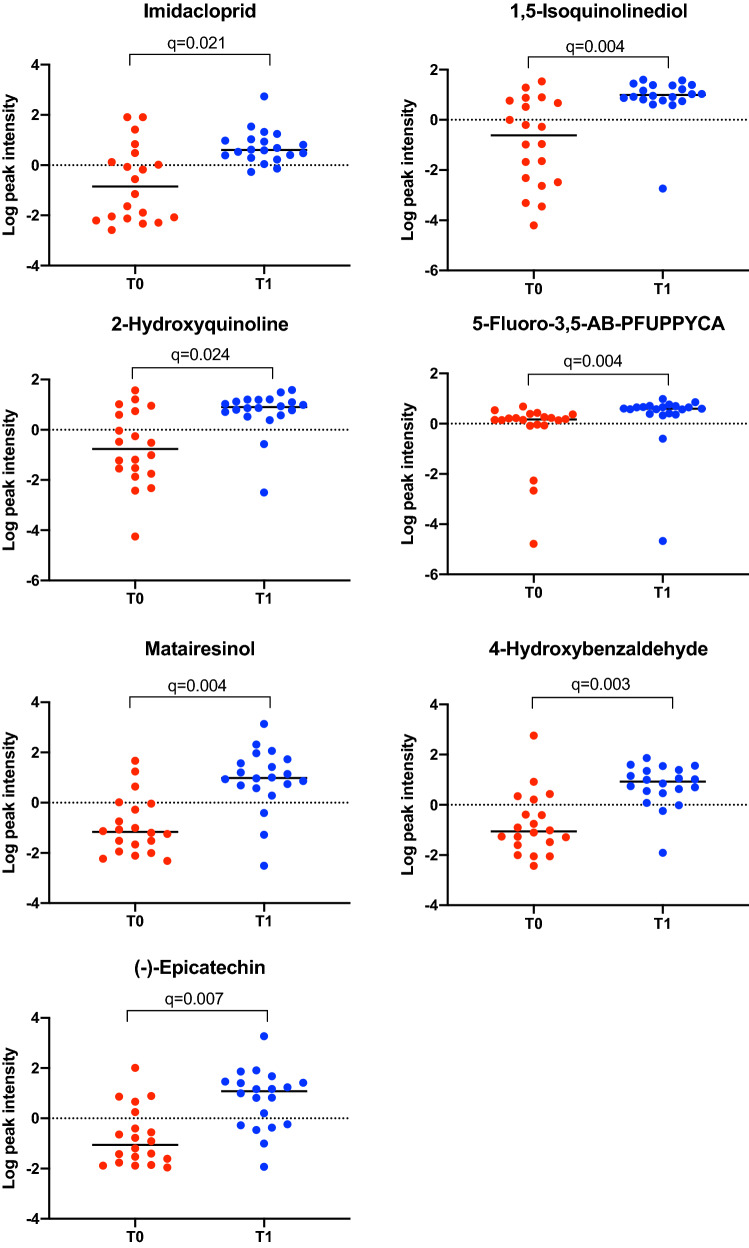
Normalized data were used to create individual value graphs of the fecal metabolites that increased after weight reduction in dogs. The bar represents the median for each group, (T0) before, (T1) after a period of weight reduction. Wilcoxon tests were used for statistical comparison and significance was based on FDR values

Purine, 1-methylxanthine, cetrimonium, coumestrol, trigonelline, and L-(-)-methionine decreased after weight loss (Fig. 2). Conversely, imidacloprid, 1,5-isoquinolinediol, 2-hydroxyquinoline, 5-fluoro-3,5-AB-PFUPPYCA, matairesinol, 4-hydroxybenzldehyde and (-)-epicatechin increased after weight loss (Fig. 3).

**Fig. 4:**
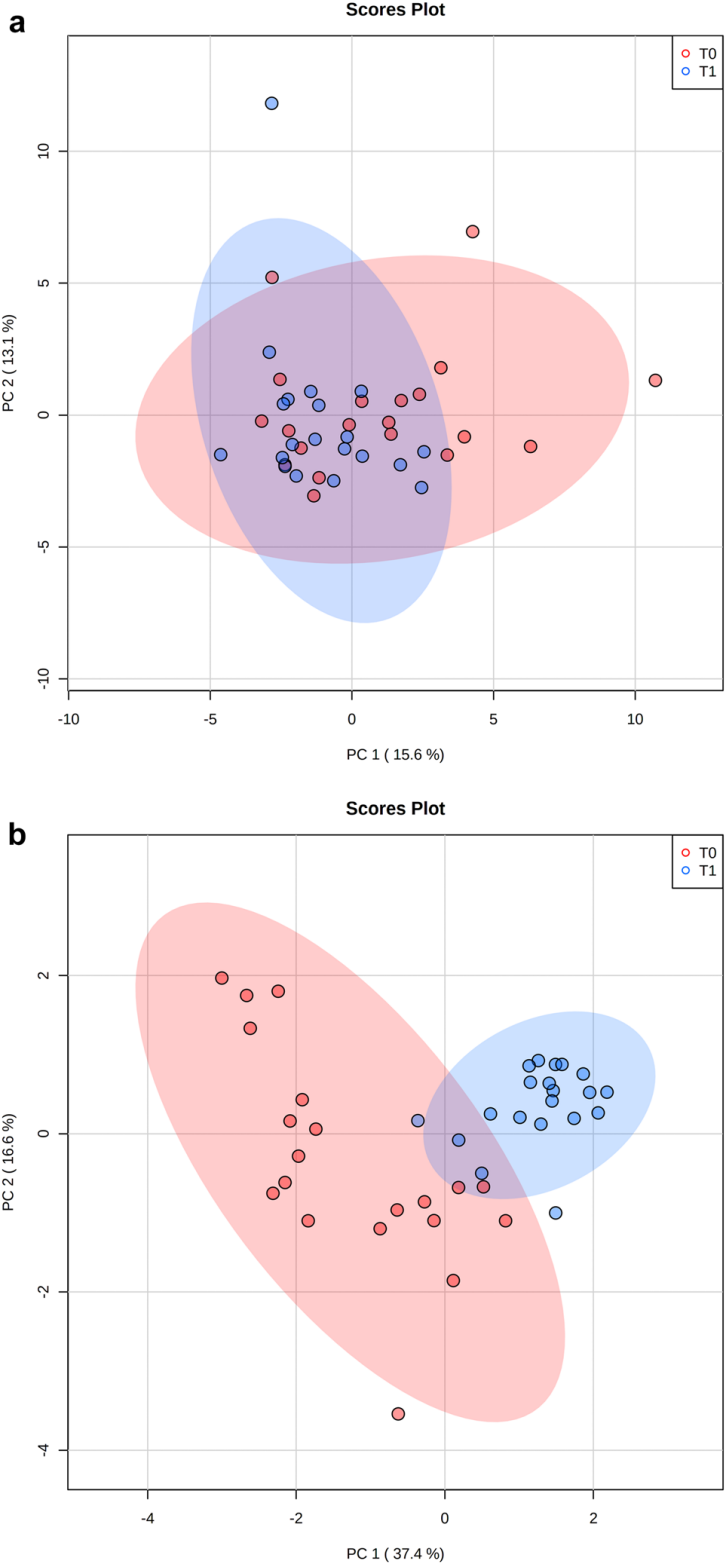
**A** 2D PCA score plot displaying the fecal metabolomics data from dogs before (T0, red) and after (T1, blue) weight reduction by feeding a therapeutic diet. **B** 2D PCA score plot displaying the data from the 13 metabolites which were significantly different in their peak intensity before (T0, red) and after (T1, blue) weight loss reduction. Ellipses represent the 95% confidence interval of the metabolite profile for each group

Based on PCA, there was no separation of the overall metabolic profile of dogs before (T0) and after (T1) weight reduction (Fig. 4A). However, clear separation was observed on the PLS-DA plot (Supplementary Fig. 1). In addition, when the 13 compounds which showed a significant different concentration after weight loss were plotted on the PCA, separated clusters were shown for each group of dogs (Fig. 4B).

## Discussion

In this study, we investigated the fecal metabolome of obese dogs following a controlled weight reduction program that utilized a diet rich in fiber and protein. In a previous study involving this cohort, significant changes in the composition of the fecal microbiome composition had been observed after weight reduction (Bermudez Sanchez et al., 2020). However, given that many different bacterial species can occupy the same niche, changes in microbiome composition do not necessarily result in changes in microbiome function. Therefore, to understand whether the changes in the fecal metabolome may have an impact on intestinal health, we chose to analyze the fecal metabolome of the same twenty obese dogs before and after weight loss in the current study. Overall, we were able to show significant changes in the concentration of 13/134 fecal metabolites. Whilst notable, the magnitude of these differences was insufficient to create a separation in the metabolome profiles on PCA plot (Fig. 4A). The reason for this is not known, but might be related to the variety of diets that dogs were fed before the start of the study, and this variability might have obscured the shifts seen with weight reduction as a result of the therapeutic diet fed. Furthermore, the dogs studied were diverse in terms of their signalment and, being pet dogs, were exposed to different environmental factors. Additional work would be required to examine this possibility further.

The specific changes seen in the fecal metabolome profile in the current study include decreases in the concentrations of purine, 1-methylxhantine, cetrimonium, coumestrol, trigonelline, and L(-)-methionine, and an increase in the concentrations of imidacloprid, 1,5-isoquinolinediol, 2-hydroxyquinoline, 5-fluoro-3,5-AB-PFUPPYCA, matairesinol, 4-hydroxybenzaldehyde, and (-)-epicatechin (Figs. 2 and 3).

Purine is a metabolic substrate of intestinal bacteria, which has been reported to be high in people ingesting high-protein diets (Guo et al., 2016). Thus, a decrease in purine concentration is contrary to expectations, given that the therapeutic diet fed was rich in protein. Possible reasons for this include the reduced food intake during the weight reduction period, an increase in purine requirement by the host, or differences in the composition of the microbiota, leading to a change in the metabolic syntrophy of the bacteria (Bermudez Sanchez et al., 2020). This has been observed for the alkaloids 1-methylxanthine, which is a substrate of different species of *Pseudomonas* (Woolfolk & Downard, 1977; Yu et al., 2009), and trigonelline, which modulates the intestinal microbiota in animal models (Anwar et al., 2018; Bruce-Keller et al., 2020), and plays a role in the metabolism of choline, indirectly affecting lipid accumulation as well as improving specific markers of health status during ingestion of a high-fat diet in mice (Knott et al., 2017; Salek et al., 2007; Zhou et al., 2012).

Another compound that decreased in fecal samples after weight loss was the amino acid L-(-)-methionine, which is usually present in the diet of dogs (Harrison et al., 2020; Middelbos et al., 2006). It has been reported that an increased intake of this amino acid is associated with adverse effects on host metabolism and the gut microbiome in mice (Miousse et al., 2017). Therefore, dietary methionine restriction has a protective role on health, extending life expectancy, reducing the weight gain, and regulating insulin in mice (Ables et al., 2012; Yang et al., 2018). Conversely, methionine supplementation is associated with improved intestinal integrity and oxidative status in piglets (Su et al., 2018; Yang & Liao, 2019). Interestingly, L-(-)-methionine is a substrate of gut bacteria, including members of the Proteobacteria phylum, the Bacilli class and the *Clostridium* and *Bifidobacterium* genera (Portune et al., 2016).

Dietary polyphenols are bioactive compounds with antioxidant activity, and are commonly found in fruits, vegetables, seed, and beverages such as coffee, tea, and wine (Cardona et al., 2013). Polyphenols in fecal samples arise directly from the diet; however, some polyphenols are also subject to metabolism by gut bacteria (Gaya et al., 2016), as is the case for (-)-epicatechin (Tzounis et al., 2011). Consumption of polyphenols has been associated with a modulation of the intestinal microbiota (Lee et al., 2006), maintenance of host intestinal barrier integrity, and regulation of immune functions (Kolátorová et al., 2018). Potential anti-obesogenic effects have also been attributed to some polyphenols, including catechins (Wang et al., 2014), with possible mechanisms including suppression of fat oxidation, inhibition of adipocyte differentiation, inhibition of fat absorption, and induction of adipocyte apoptosis (Hursel & Westerterp-Plantenga, 2010; Lin et al., 2005). Intestinal bacteria can also transform matairesinol into mammalian lignan enterolactone, which can undergo colonic absorption (Jansen et al., 2005) and is also known to possess estrogenic, and antioxidative functions (Borriello et al., 1985; Bowey et al., 2003; Heinonen et al., 2001). In the current study, various polyphenolic compounds were detected in the fecal samples of dogs with obesity. Given that (-)-epicatechin and matairesinol concentrations were increased, but coumestrol concentration was decreased, our results did not demonstrate a consistent effect of weight reduction on the fecal polyphenolic profile in dogs with obesity. Further studies would be required to determine the significance of these findings.

Concentrations of quinolines 2-hydroxyquinoline and 1–5, isoquinolinediol decreased after weight reduction in dogs with obesity. Quinoline derivates are products of bacterial metabolism with quorum-sensing functions (Hubbard et al., 2019) that can be metabolized by some bacteria, such as *Rhodococcus* sp., *Pseudomonas* (Sun et al., 2009), *Thauera*, and *Bacillus* (Tuo et al., 2012). Interestingly, in humans, 2-hydroxyquinoline inhibits PON1 (paraoxonase-1), an antioxidant enzyme involved in lipid metabolism (Graves & Scott, 2008). Furthermore, some quinoline derivates have been proposed as Aryl hydrocarbon receptor (AhR) ligands in humans (Hubbard et al., 2019). AhR signaling contributes to the immune homeostasis by suppressing inflammation but also modulating the maturation of immune cells involved in the mucosal immunity and the defense against pathogens by xenobiotic metabolism and epithelial renewal in the intestine (Agus et al., 2018; Lamas et al., 2018; Stockinger et al., 2014; Zhou, 2016).

We also detected changes in the concentrations of the synthetic compounds imidacloprid, cetrimonium, and 5-fluoro-3,5-AB-PFUPPYCA. Imidacloprid is an antiparasitic drug widely used in companion animals (Jeschke & Nauen, 2008), but it is also used as an insecticide in agriculture (Badgujar et al., 2013). Administering this compound together with a high fat diet is associated with greater adiposity and insulin resistance in mice (Sun et al., 2016), but the effects in dogs have not previously been reported. Cetrimonium is a disinfectant whereas 5-fluoro-3,5-AB-PFUPPYCA is a synthetic cannabinoid derivative but, to our knowledge, the relevance of the changes in the concentration of the rest of these synthetic compounds after weight loss and during health in general has not yet been studied.

A limitation of the current study is that it is unclear whether the observed changes in the fecal metabolome were the result of the weight reduction, or whether they were simply the result of the dietary change, as demonstrated by a recent study on the fecal metabolome of dogs (Jackson & Jewell, 2019). A second limitation was that only two fecal samples were collected from each dog, meaning that sequential changes in the intestinal microbiome could not be elucidated. This was further complicated by the fact that follow-up samples were taken after a variable time, dictated by the duration of weight loss, rather than being taken at specific time points after the start of the study.

In conclusion, although preliminary, the findings of the current study suggest that controlled weight reduction in obese dogs using a protein and fiber rich therapeutic diet is associated with alterations in the fecal metabolome, characterized by increased concentrations of polyphenols and quinoline derivatives. However, further studies are necessary to clarify the significance of these findings, and if they are a consequence of the therapeutic diet, a direct effect of weight reduction, or are an indirect effect of changes in the composition of the fecal microbiome.

## Supplementary Information

Below is the link to the electronic supplementary material.

Supplementary data 1. Spreadsheet of the data from peak intensity for each sample and compound imported from Compound discoverer 2.1. (XLSX 341 kb)

Supplementary data 2. Association between baseline fecal metabolites and sex, and between baseline fecal metabolites and age. (XLSX 102 kb)

Supplementary figure 1. 2D PLS-DA score plot displaying the fecal metabolomics data from dogs before (T0, red) and after (T1, blue) weight reduction by feeding a therapeutic diet. Ellipses represent the 95% confidence interval of the metabolite profile for each group. (PNG 213 kb)

## Data Availability

The metabolomics data reported in this paper are available via supplementary material.
