# Supplementary figures and images for: Untargeted fecal metabolome analysis in obese dogs after weight loss achieved by feeding a high-fiber-high-protein diet
Source: Metabolomics. 2021 Jul 6;17(7):66. doi: 10.1007/s11306-021-01815-1 (PMC8260550; doi:10.1007/s11306-021-01815-1)

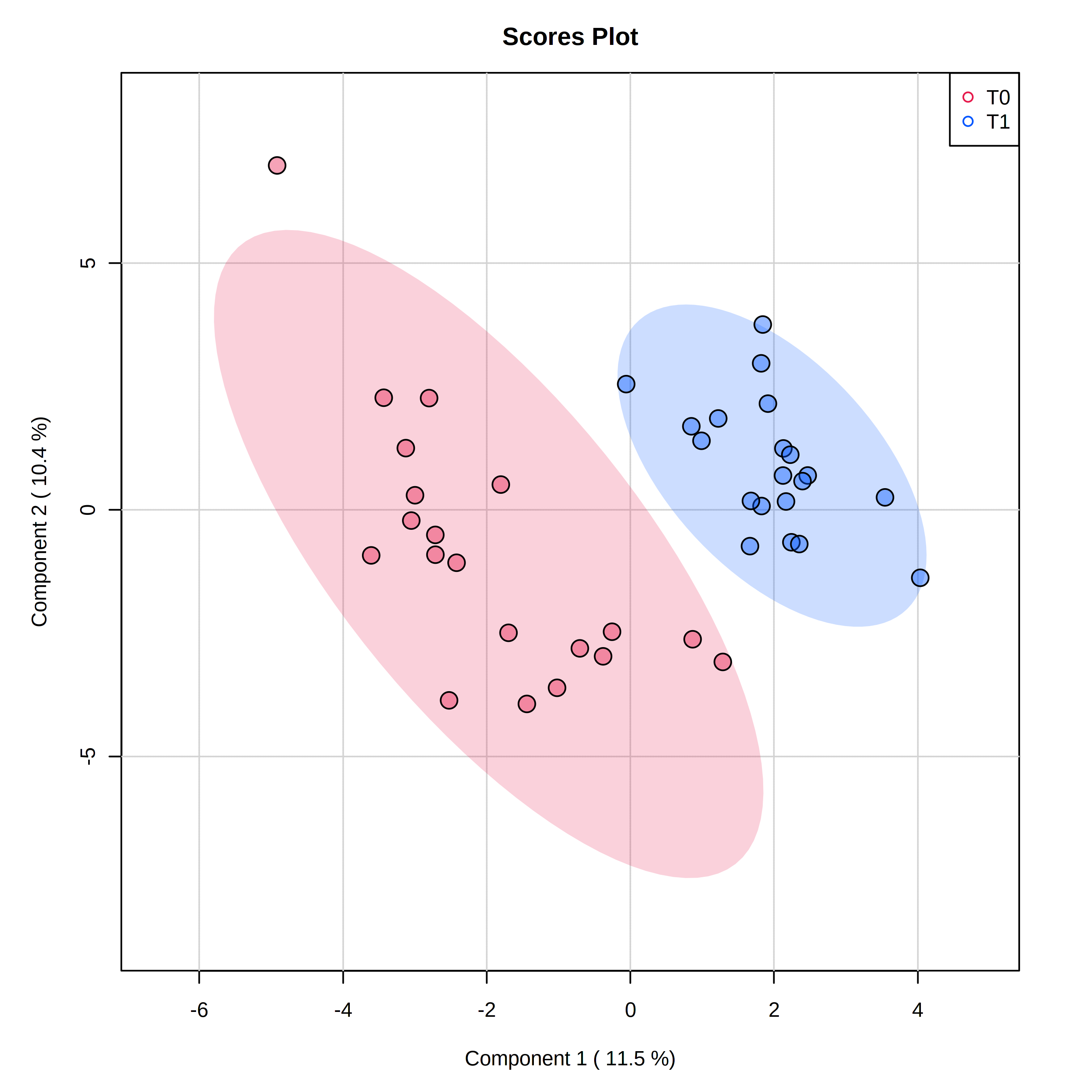

Supplement: Supplementary file 3 — Supplementary figure 1. 2D PLS-DA score plot displaying the fecal metabolomics data from dogs before (T0, red) and after (T1, blue) weight reduction by feeding a therapeutic diet. Ellipses represent the 95% confidence interval of the metabolite profile for each group. (PNG 213 kb) [file 11306_2021_1815_MOESM3_ESM.png]
